# 

**Published:** 1908-04

**Authors:** 


					﻿CONTENTS.
COMMUNICATIONS—
the Treatment of Bright’s Disease. By H. F. Harris, M. D., Atlanta, Ga.-......——
/alue of Determining the Opsonic Index in the Treatment of Chronic Infections With Vaccine^.
By J. Edgar Paullin, B. A., M.D..................................................—-
Jbsevtions in 271 cases of Mastoiditis. By F. P. Calhoun...........................>—*
Excesses of Modern Times and Their Relation to Disease. By Marion Me. H. Hull, M. D., Atlanta. Ga— —-1
Address of C. B. Boothe, President of the California Association for the Study and Prevention of Tuber-
culosis, at Annual Meeting, December 3,1907 .....................................  —.2)
Hyposspodias—Pseudohermaphrodite. By Edgar G. Ballenger, Atlanta. Ga........................—-2S
Spring Coughs. By James Burke, M. D., Mantowac, Wis...........................  —	.....31
CORRESPONDENCE........................................................................ S'-
EDITORIAL NOTES AND COMMENTS—
Evolution and the Children of Freedom..............................................
Inherited Syphilis..........................................................................	—
Cancer Research_-.....................................................................
Urotropin Excreted in the Bile and Pancreatic Juice...................................
NEWS AND NOTES..........................................................................-
BOOK REVIEWS......................................................................   ...
SELECTIONS AND ABSTRACTS..............................................................---
				

